# Resizing Approach to Increase the Viability of Recycled Fibre-Reinforced Composites

**DOI:** 10.3390/ma13245773

**Published:** 2020-12-17

**Authors:** Vsevolod Matrenichev, Maria Clara Lessa Belone, Sarianna Palola, Pekka Laurikainen, Essi Sarlin

**Affiliations:** Engineering Materials Science, Tampere University, FI-33014 Tampere, Finland; acrossq@gmail.com (V.M.); clara.lessabelone@tuni.fi (M.C.L.B.); sarianna.palola@tuni.fi (S.P.); pekka.laurikainen@tuni.fi (P.L.)

**Keywords:** glass fibres, carbon fibres, recycling, surface treatments

## Abstract

Most recycling methods remove the essential sizing from reinforcing fibres, and many studies indicate the importance of applying sizing on recycled fibres, a process we will denote here as resizing. Recycled fibres are not continuous, which dissociates their sizing and composite lay-up processes from virgin fibres. In this study, commercial polypropylene and polyurethane-based sizing formulations with an aminosilane coupling agent were used to resize recycled glass and carbon fibres. The impact of sizing concentration and batch process variables on the tensile properties of fibre-reinforced polypropylene and polyamide composites were investigated. Resized fibres were characterized with thermal analysis, infrared spectroscopy and electron microscopy, and the tensile properties of the composites were analysed to confirm the achievable level of performance. For glass fibres, an optimal mass fraction of sizing on the fibres was found, as an excess amount of film former has a plasticising effect. For recycled carbon fibres, the sizing had little effect on the mechanical properties but led to significant improvement of handling and post-processing properties. A comparison between experimental results and theoretical prediction using the Halpin-Tsai model showed up to 81% reinforcing efficiency for glass fibres and up to 74% for carbon fibres.

## 1. Introduction

The manufacturing and use of glass and carbon fibre-reinforced composite materials based on thermoset polymers have increased during the last decades in all engineering applications. Naturally, the amount of end-of-life (EoL) composites has been increasing as well. A common approach to deal with EoL fibre-reinforced composites has been landfilling, although it is the least preferred option for waste management, as stated in the European Waste Framework Directive [1]. Therefore, some European countries have already banned the landfilling of composites. From a circular economy point of view, the best alternatives to landfilling would be recycling and reusing.

Much work remains to make the recycling of composites viable in industrial scale. Several methods, such as thermal [2,3], steam [4], electrochemical [5], microwave induced [6] and mechanical [7] recycling, have been used to recover reinforcing materials from composite waste. The recycling processes are constantly developed, and some commercial recycled fibre products are available [8,9]. However, in most of the recycling processes, the physical properties, surface quality and origin of the recycled fibres cannot be fully controlled leading to non-uniform and degraded fibre properties, which downgrades the further potential application possibilities of the recovered fibres [10].

Most of the recycling processes remove the sizing layer from the fibre surface [11]. Sizing is a thin multicomponent coating applied on the fibres during manufacturing to ease processing and improve the performance of the composite. In composite materials, the sizing layer creates an interphase at the fibre-matrix interface, which thickness can range from 30 to 200 nm [12,13]. The sizing formulation consists mainly of organic components and the choice of the sizing components depends on the material and geometry of the fibre and the intended matrix [14]. The exact composition of the sizing on commercial fibres are company secrets of the fibre manufacturers [15], but the sizing solutions are typically water-based and the most important components for the performance are the film former and, or glass fibres, the coupling agent [16,17]. The main role of the film former is to provide good processing characteristics, protection against fibre-fibre damage during transportation and handling and bond the fibres into a bundle. The film former is also important for optimal interfacial properties. The coupling agent is responsible for increasing the fibre-matrix adhesion having two types of functional groups, one of which is able to bond with the inorganic glass fibre surface and the other with the organic matrix [18]. The coupling agent might also bridge minor defects on the glass fibre surface [15]. 

The application of sizing on recycled fibres, denoted here as “resizing”, could help to bridge the gap between virgin and recycled fibres by mitigating the issue of uneven fibre properties and by guaranteeing a proper fibre-matrix adhesion. This is also acknowledged in the current literature [18,19], although no data are available about the optimization of the sizing composition or processes for recycled fibres. In this study, the aim was to understand the effect of the applied resizing batch process variables on the processing and properties of the composite. Thermally recycled chopped glass and carbon fibres were sized and compounded with different thermoplastic matrices. Thermoplastic composites were seen as a potential application area of recovered fibres considering the decreased fibre properties during thermal recycling. Fibres were characterized after the resizing process with thermal analysis, infrared spectroscopy and electron microscopy to understand the effect of the processing parameters. The tensile properties of the thermoplastic compounds were analysed to confirm the achievable level of performance and compared with a theoretical ideal value to evaluate the reinforcing efficiency of the resized fibres.

## 2. Materials and Methods 

### 2.1. Materials

**Figure 1:**
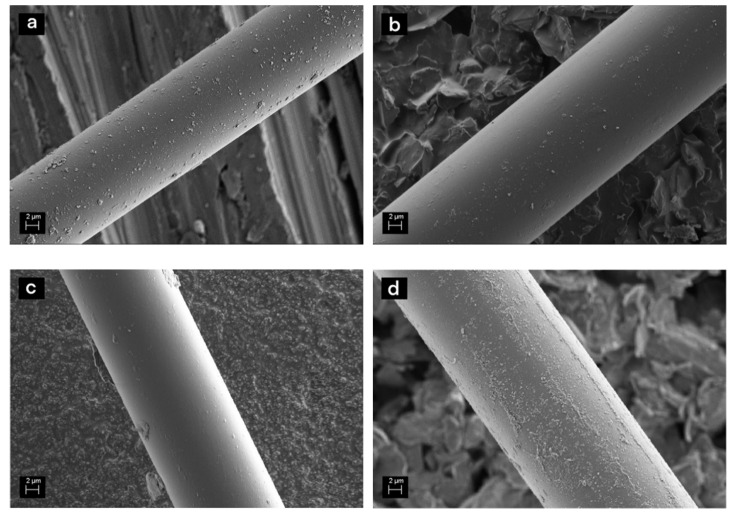
SEM images of (**a**) rGF before sizing, (**b**) rGF with 1 w-% PP sizing (rinsed), (**c**) rGF with 5 w-% PP sizing (rinsed) and (**d**) rGF with 5 w-% PP sizing (without rinsing).

Thermally recycled glass fibres (rGF) from EoL wind turbine blades and carbon fibres (rCF) from EoL aerospace components were used. The fibres were recycled by TECNALIA (Basque Research and Technology Alliance, Donostia-San Sebastián, Spain) with conventional pyrolysis process. The exact origin (fibre type, manufacturer) of the recycled fibres was not known. Single fibre tensile test with the Fibrobotics device [19] (Fibrobotics, Tampere, Finland) was used to characterize the performance of the recycled fibres (Table 1). It is highly probable that the fibres were degraded due the use and pyrolysis, and therefore, the properties were low and scatter high compared with pristine fibres. In total, 50 fibres were tested to calculate the tensile properties for both materials with a gauge-length of 23.5 mm and crosshead velocity 0.008 mm/s. The diameter of each individual fibre was measured through with the optical setup of the Fibrobotics device (Fibrobotics, Tampere, Finland). Micrographs of recycled fibres are shown in Results (Figures 1a and 5a).

In general, the selection of a film former and a coupling agent depends on the type of matrix to be used. In this study, the resized glass and carbon fibres were compounded with heterophasic polypropylene copolymer (PP, tensile modulus of 1300 MPa) and polyamide 6 (PA6, tensile modulus 2300 MPa). Due to the confidential rules of the related project, more detailed information about the matrices could not be provided, but the analysis was based on a comparison between neat resin and composites. A non-ionic malleated polypropylene dispersion (Hydrosize^®^ PP2-01, Michelman Inc., Cincinnati, OH, USA) was used with the PP matrix as the film former. It improves the interfacial properties by bonding with the fibre surface and/or the coupling agent (in the case of rGF) and mixing well with the PP matrix. The film former is designed for both chopped strands and continuous fibres to provide enhanced impact resistance, slip and lubricity. According to the supplier, the concentration of the non-volatile part of the solution is 38.5–41.0%. A non-ionic polyurethane dispersion (Hydrosize^®^ U2022, Michelman Inc., Cincinnati, OH, USA) was used as film former with the PA6 matrix. The dispersion is designed for chopped strands, and it provides good impact resistance, thermal stability and adhesion with the matrix. According to the supplier, the concentration of the non-volatile part in the solution is 58–60%. Both film formers are informed to be compatible with glass and carbon fibres. For the sizing of rGF, 3-aminopropyltriethoxysilane (99% purity, Sigma-Aldrich, St. Louis, MO, USA) was chosen as the coupling agent for both matrices. Acetic acid (Sigma-Aldrich, St. Louis, MO, USA) was used to hydrolyse the coupling agent functional groups and to control the pH of the sizing solutions. The chemicals were mixed with deionised water in the sizing solutions. 

### 2.2. Resizing Process

During virgin fibre manufacturing, GFs and CFs continuously proceed to the sizing applicator immediately after the fibre formation, and the sizing time is commonly less than 1 ms. In the case of chopped recycled fibres that are in a tangled cluster, a batch-type dipping process must be used. The sizing process time must be increased to allow the wetting of all fibres in the batch. 

For rGF, the coupling agent, water and acetic acid were mixed for 1 h to hydrolyse the alkoxy groups of the organosilane coupling agent. This enables the formation of siloxane bridges and hydrogen bonded silanol linkages between the fibre surface and the coupling agent. The other end of the coupling agent can then bond with the film former and the matrix [17]. The film former and more water were then added, and the resulting solution was mixed for another 1 h to obtain a homogeneous mixture. On the last stage, acetic acid was added to set the solution pH to 7. The temperature of the solution during mixing was 30 °C to ensure completely homogeneous mixture. 

Solutions with 1 and 5 w-% absolute concentration of solid content were prepared. The relative concentrations of film former and coupling agent were 90 and 10 w-%, respectively, to avoid self-polymerisation reaction of the coupling agent. A high concentration of coupling agent can lead to reactions in the solvent phase instead of the silane bonding with the fibre surface [18]. Consequently, a significant amount of the sizing could be removed from the fibres surface during the rinsing stage or while handling, since it is not chemically bonded to the fibres. This would likely also lead to poor interfacial properties. The exact recipe of the sizing solution is given in Table 2. The rGFs were immersed into the solution for 30 s. A part of the fibres was subsequently rinsed with distilled water to remove the excess of sizing, while this step was omitted for the rest of the fibres to study the effect of the rinsing. On the last step, the fibres were dried in a convection oven at 80 °C for 10–12 h to remove the moisture and kept in plastic bags before compounding.

For the rCF, two types of pre-treatment options were used prior to sizing: acetone washing and acidic treatment. In the acetone washing, the rCFs were sonicated in acetone for two consecutive 2 min cycles. The solvent was changed between the cycles. After treatment, the fibres were dried in a convection oven at 80 °C for 12 h. The objective of the acetone treatment was to evaluate the need for washing the fibres prior to compounding and/or resizing to remove a possible weak residual carboneous layer, which can form on the rCF surface during recycling [20]. In the acidic treatment, the rCFs were soaked in a bath of 65% HNO_3_ at 60 °C for 20 min. The fibres were then rinsed thoroughly with deionised water until the pH of the rinsing water was neutral indicating that no acid was removed by the rinsing anymore. The fibres were dried in a convection oven at 80 °C for 12 h. The purpose of the acid treatment was to oxidize the rCF surface and provide reactive side groups that could react with the matrix [15] and, thus, improve the fibre-matrix adhesion. The effect of the pre-treatments was studied only with the PP film former.

For the rCF resizing, two concentrations of each film former were prepared: 1 and 5 w-% solids content with deionised water. Each of the solutions was heated up to 30 °C and kept at this temperature for 1 h. The rCFs were immersed into each of the solutions and then rinsed with deionised water to remove excess sizing. Finally, the fibres were dried in a convection oven at 80 °C for 10–12 h and sealed in plastic bags before compounding. The sizing formulation and process description for the rCF samples are shown in Table 3.

### 2.3. Compounding

The rGFs and rCFs were compounded with the PP and PA6 matrices in a laboratory-scale twin-screw microcompounder (Xplore, Sittard The Netherlands). A screw rotation rate of 80 min^−1^ was used, the processing temperatures were 190 °C for PP matrix and 230 °C for PA6 matrix. The compounding time was 1 min. The material was formed into dog-bone-shaped tensile specimens (ISO 527-2 Annex A, sample type 1BA [21]) with a microinjection unit (Xplore, Sittard, The Netherlands). Prior to the compounding, the polymer granulates were dried in vacuum for 12 h at 80 °C to remove moisture that could affect the processing. Both fibres were chopped to approximately 5 mm length before compounding.

The target fibre fraction in the compounds was 20 w-%. This was verified by determining the fibre weight fraction of the tensile test specimens with the burning-off method (ISO 1172, Method A [22]). For the rCF, nitrogen atmosphere was used. The specimens were heated up to 500 °C to remove the polymer matrix.

### 2.4. Analysis Methods

The morphology of the fibres and fracture surfaces of the tensile test specimens were analysed by scanning electron microscopy (SEM, Zeiss ULTRAplus, Oberkochen, Germany). The samples were coated with a thin carbon coating to enhance their conductivity.

Thermogravimetric analysis (TGA, Netzsch TG 209 F3 Tarsus, Selb, Germany) measurements were performed for resized fibres to evaluate the amount of sizing on the fibres. Temperature range was from 30 to 700 °C and the heating rate 10 K/min in a nitrogen atmosphere.

The functional groups on the fibres were identified using Fourier-transform infrared spectroscopy (FTIR) with Bruker Tensor 27 spectrometer (Ettlingen, Germany) using a PIKE Technologies (Madison, WI, USA) GladiATR Attenuated Total Reflectance (ATR) sample holder. The ATR material was a diamond crystal. The wavenumber range of analysis was 600–4000 cm^−1^. For coupled TGA-FTIR measurements, the ATR sample holder was replaced with an internal TGA-FTIR coupling accessory (Bruker, Ettlingen, Germany), and the same TGA device was used as above. The measured wavenumber range was 600–4500 cm^−1^.

The tensile properties of the composite specimens were tested with Instron 5067 universal testing device (Instron, Norwood, MA, USA) with a 30 kN load cell. The tension tests were carried out at a strain rate of 10 mm/min; ten specimens were tested for each sample.

## 3. Results and Discussion

### 3.1. Resizing of rGF

Matrix residues on the fibre surface after recycling can significantly hinder the functionality of the new sizing, decreasing adhesion between the matrix and the fibres. SEM analysis prior to the sizing (Figure 1a) revealed some residues of matrix and original sizing on the rGF surfaces, which were also assumed to be present in the resized fibres. However, the amount was small, and additional cleaning steps were not considered necessary. This was also supported by the TGA and FTIR results shown later. No other surface defects or presence of matrix between the fibres could be detected. Figure 1b–d reveal that the sizings covered the rGF surface and most of the residues and formed smooth layer, similar to sizing layers on virgin glass fibres. However, slight agglomeration of the sizing could be detected on the surfaces of the fibres sized without rinsing (Figure 1d).

**Figure 2:**
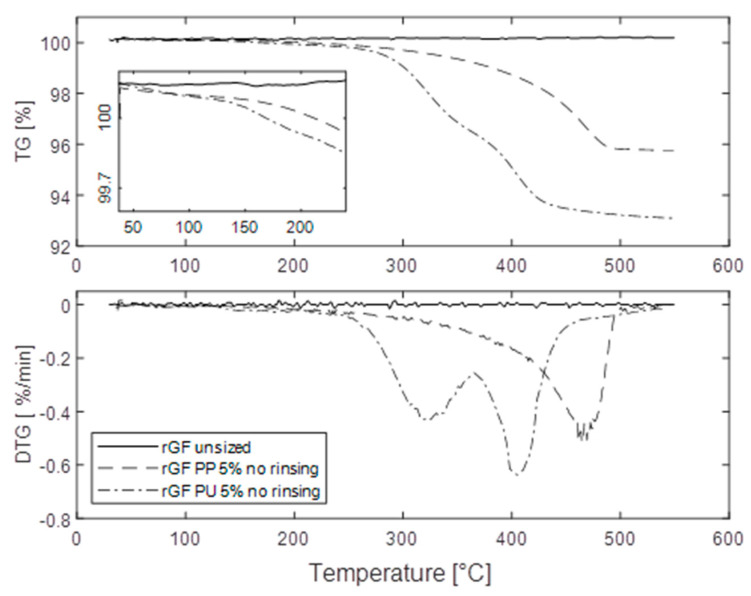
Examples of the rGF TG curves together with their derivatives (DTG curves) before sizing and after 5 w-% PP and PU sizing (without rinsing).

Glass is assumed to be stable at the temperature range of the thermal analysis (TGA) and the observed mass changes originate from the degradation of organic materials of the sizing on the fibre [23]. Thermal desorption on the surface involves breaking bonds initiating from the weakest ones [24] and the rate of mass loss on TGA measurements can be assumed to be proportional to the rate of bond break [25]. The rGF before sizing did not show any mass loss (Figure 2), indicating the recycling process was performed adequately. Within one sizing type, the mass loss temperatures of the sized fibres were always similar, while the magnitudes of the mass losses varied. The total mass losses indicating the amount of sizing on the fibre are listed in Table 2.

The TGA curves of resized fibres represented two mass loss steps for the PP-based sizing and three steps for the PU-based sizing (Figure 2). The first step took place at the same temperature (100–200 °C) and was very small for both sizing types. In addition to the possible residual moisture on the fibres, this step can be attributed according to Nagel et al. [26] to the elimination of water from the condensation of Si–OH caused by the decomposition of surface bonded coupling agent and to the desorption of carbon dioxide from carbamate originated from the interaction with silane amine group. For both sizing types, the second mass loss step indicates desorption and degradation of the sizing. Within this mass loss step, the weakly bonded fractions, the stronger physical interactions and possible chemical bonds between the sizing components and the fibre surface are broken [13]. The sizing has multiple ways to interact with the fibre surface, which may lead to the establishment of bonds of dissimilar energies. This way, the activation energy required to rupture them varies and desorption occurs in a broad region, as observed here. With increasing temperature, the bonds of the sizing layer break resulting in smaller, volatile molecular fragments, while the heavier ones remain on the surface in the solid or liquid phase for some time until their volatilization [27]. The third step for PU-sized fibres is related to the degradation of the PU film former.

**Figure 3:**
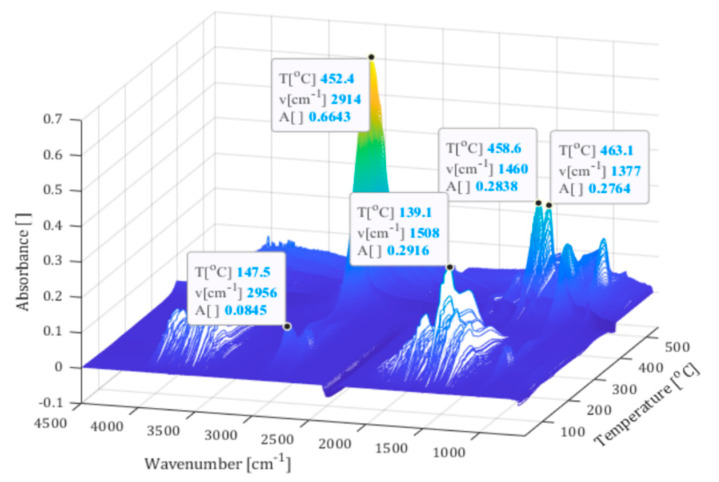
Results of the TGA-FTIR analysis for the PP film former solution.

Further understanding of the nature of the mass loss steps was achieved by a coupled TGA/FTIR measurement from the film former solution. A 3D plot of the results is presented in Figure 3. The TGA curve from this measurement corresponded well with the TGA curve from the sized fibres. The FTIR analysis indicated that the initial mass loss was mainly water with a relatively small concentration of hydrocarbon species, most likely some low molecular mass film former molecules. The FTIR detected these absorption bands to temperatures up to 300 °C. The spectrum corresponding to the latter mass loss, between 300 and 480 °C, had the characteristic absorption bands of polypropylene, indicating evaporation of the film former itself.

As expected, rinsed rGF samples showed significantly lower mass losses compared to samples without rinsing (Table 2), but the differences between different sizing solution concentrations were not as systematic. In the case of PP-based sizing, higher sizing solution concentration led to higher sizing desorption for rinsed samples whereas in the case of PU-based sizing, the mass losses were equal for different concentrations. Malleated PP is able to form bonds not only with the coupling agent but also with the fibre surface [28]. Therefore, increasing the concentration of the sizing components could increase the amount of the sizing on the surface even after rinsing. Such bonding is unlikely for the PU film, which can only weakly bond to the glass surface via hydrogen bonding. Therefore, the amount of strongly bonded sizing is dependent on the coupling agent, and the rinsing is expected to remove most of the excess sizing from the surface [29]. Without rinsing, the sizing also acts as a binder between the individual fibres. However, increasing the sizing solution concentration did not affect the amount of PP-based sizing, whereas for PU-based sizing the amount of sizing increased. This might be indicative of the relative strengths of the internal interactions of the sizing components, which can be assumed stronger for the PU-based sizing.

**Figure 4:**
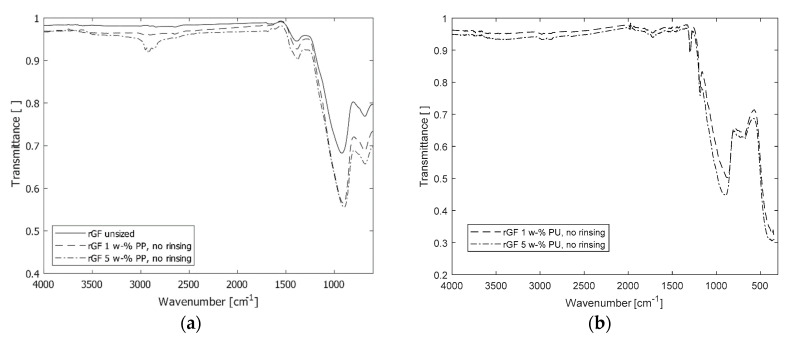
The FTIR spectra of the rGF samples before sizing and after 1 and 5 w-% PP sizing (**a**) and PU sizing (**b**) solutions.

Examples of FTIR spectra of rGF are presented in Figure 4. The shoulder type region between 600 and 1200 cm^−1^ is the main noticeable detail of the spectra. This predominant area is associated to the different vibrational modes of Si–O and Al–O bonds and combination vibrations in the glass structure [27]. The medium intensity peak at 1400 cm^−1^ is related to the stretching vibrations of BO_3_ groups that are present in the E-glass structure [30]. For the sized samples, the 600 and 1200 cm^−1^ region were stronger compared to the fibres before sizing. Although it is not possible to differentiate between Si bonds coming from rGF or coupling agent, this increase can be attributed to the presence of sizing in the fibre surface, because the bulk fibre remains unchanged during the resizing process. Another difference between the fibres with PP sizing and without sizing are the areas of 2800–3000 and 1300–1500 cm^−1^. The first one is assigned to the stretching vibration of C–H bond in CH_2_ and CH_3_. The sharp shoulder in the 5 w-% sample at 1374 cm^−1^ corresponds well to the CH_3_ “umbrella” vibration. Although the peak intensities are weak, these absorption bands can be attributed to the presence of film former, due to its PP backbone [31], and of coupling agent, due to its aminopropyl segment [32]. The changes in the intensities of the C–H vibrations were noticeable in most of the sized samples and increased with increasing sizing solution concentration. The observable IR absorption in the range of 1300 to 1500 cm^−1^ can be assigned to the bending vibrational mode of C-H of the PP backbone in malleated PP [31] and of N-H in the amine group of coupling agent [32]. For the rGF before sizing, the weak intensity in these regions indicates that most of the sizing and resin residues were effectively removed in the recycling process. For the PU-sized rGF, the difference with rGF without sizing are the areas of 1250–1350 and 1600–1750 cm^−1^. The first area and the peak at 1180 cm^−1^ are assigned to the stretching vibration of C–O bond of carboxylic group of PU film former. The second area related to C=O stretching vibration of the film former [33].

### 3.2. Resizing of rCF

**Figure 5:**
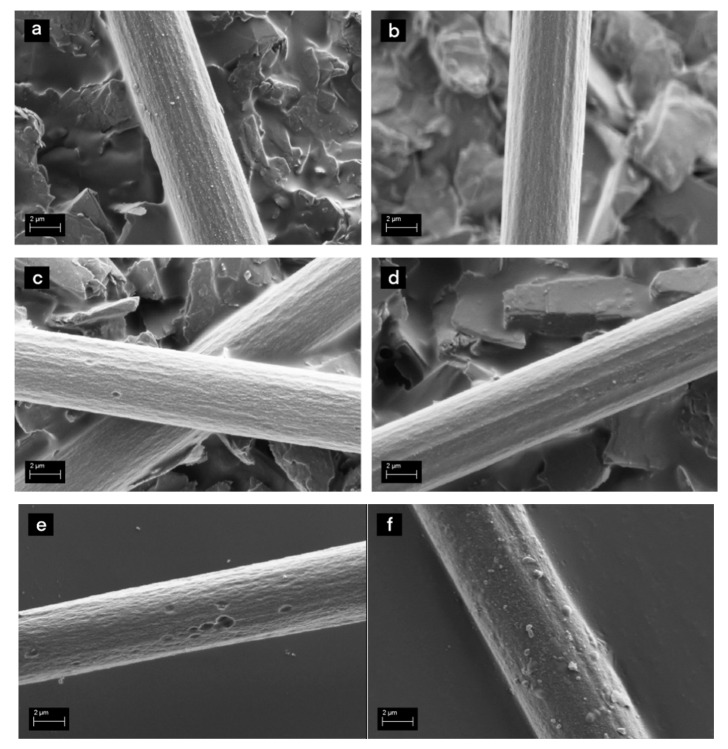
SEM images of rCF: (**a**) rCF without surface treatments, (**b**) rCF after acetone washing, and (**c**) rCF after acidic pre-treatment, (**d**) of resized rCF in PP film former with 1 w-% solution, (**e**) 1 w-% solution on acid treated rCF and (**f**) 5 w-% solution.

The effects of the acetone and acidic pre-treatments can be seen in Figure 5. The acetone-washed fibres (Figure 5b) have a smoother surface structure compared to rCF (Figure 5a). All the loose particles and impurities were removed from the surface. On the contrary, the acid treatment created an even rougher surface structure with the inclusion of some pitting (Figure 5c). The pitting may be a sign of overly harsh conditions for the fibres. The distinctive parallel grooves were also removed suggesting that oxidation of the surface has reached deeper into the fibre.

Examples of the resized rCF are shown in Figure 5d–f. The 1 w-% PP solution created a smooth and uniform coverage on the rCF, reducing the visible surface roughness (Figure 5d). For the fibre after acidic pre-treatment, a similar layer was present, but the pitting caused by the acid was visible underneath the sizing (Figure 5e). A definite increase in the sizing amount can be observed with the sizing solution of 5 w-% (Figure 5f). The rCF sized with the PU film former showed similar results.

In nitrogen atmosphere, carbon fibres are considered stable at the temperature range of the TGA experiment and mass loss to be related to the degradation and evaporation of the sizing on the fibre [34]. The mass of the rCF samples without surface treatments remained unchanged during the analysis indicating relative purity in their as-received state. The results for the sized fibres are collected in Table 3. The mass loss steps were similar with the rGF samples.

### 3.3. Properties of the rGF Composites

**Figure 6:**
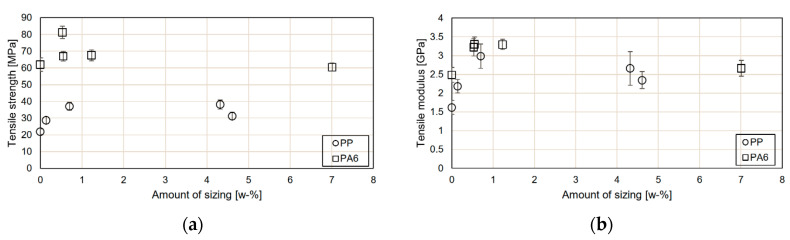
Tensile (**a**) strength and (**b**) modulus of the rGF composites as a function of the amount of sizing.

The tensile test results for the rGF-PP and rGF-PA composites are reported in Figure 6. Sizing had a clear effect increasing both tensile strength and modulus. However, it should be considered that during the sizing process the shortest fibres are lost from the batch, which affects positively on the composite properties when compared with the results without any sizing and therefore enhances the positive effect of the sizing itself. Simultaneously, this is assumed to decrease the ductility of the composites and decrease its impact performance. The stress–strain curves were similar in shape for all samples. The strain at break decreased from 9% to 3–5% for the resized samples.

**Figure 7:**
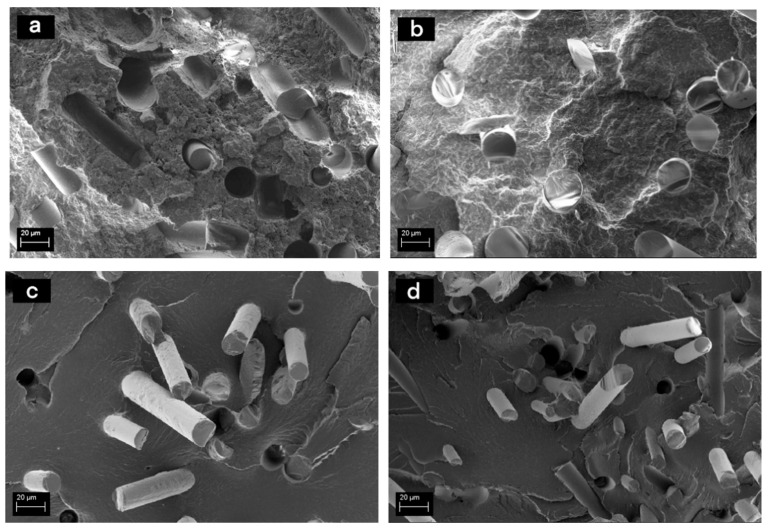
Fracture surfaces of (**a**) rGF-PP composite without fibre surface treatments, (**b**) rGF-PP composite after fibre sizing with 1 w-% PP-based sizing, (**c**) rGF-PA6 after fibre sizing with 5 w-% PU-based sizing and (**d**) rGF-PA6 after fibre sizing with 5 w-% PU-based sizing without rinsing.

The amount of sizing on the fibre surface correlated with the composite tensile properties in the case of PP showing initially an increasing trend in strength and modulus with increasing amount of sizing but at higher concentrations again a drop in the performance. This is assumed to be due to the plasticizing effect of the excess film former at the fibre-matrix interface. The fracture surface of the rGF-PP compounds (Figure 7a,b) without surface treatments was characterised with poor adhesion and interfacial contact resulting in significant number of fibre pull-outs. The sized rGF had better contact to the PP matrix.

For PA6 composites with PU-based sizing, there was no correlation between the amount of the sizing and the tensile properties (Figure 6). As the total amount of sizing was similar for the rinsed fibres from both sizing solution concentrations (approx. 0.5 w-%), but there was a significant difference in the strength of the composites, it can be assumed that the PU film former layer at the fibre surfaces is similar regardless of the sizing solution concentration, whereas the amount of coupling agent depends on its concentration in the resizing process affecting the tensile test results. This was also supported by the results described in Section 3.1. The SEM images of the rGF-PA6 fracture surfaces (Figure 7c,d) revealed that despite a seemingly good contact between the fibres and the surface with and without rinsing, there are several fibre pull-outs in the samples without rinsing. The excess film former can plasticize the interphase resulting in decreasing tensile properties, as seen in Figure 6.

### 3.4. Properties of the rCF Composites

The rCF composite tensile test results (Table 4) indicate no effect in the case of PP sizing and only a slight improvement in the case of PU sizing on the tensile strength and modulus. The strain at break was also unaffected by the amount of sizing and was approx. 5% for all rCF composites. However, the main benefit of using the sizing is, in this case, the fact that compounding is significantly easier, and a more uniform distribution of fibres can be achieved if the fibres are resized. Recycled carbon fibres without any sizing are impossible to handle and compound, as they are light and separate fibres, easily floating in the air. Resizing binds the fibres in small bundles increasing the weight of individual agglomerates and, therefore, enables compounding in industrially feasible ways. When compared with the untreated fibres, the acid treatment decreased the tensile strength, which was expected due to the observed pitting and presumptive fibre strength loss. The tensile properties of the acetone washed fibres are very similar to the fibres without washing. Considering the amount of time, chemicals and equipment needed for the washing, it would be a rather costly, slow and unnecessary process.

**Figure 8:**
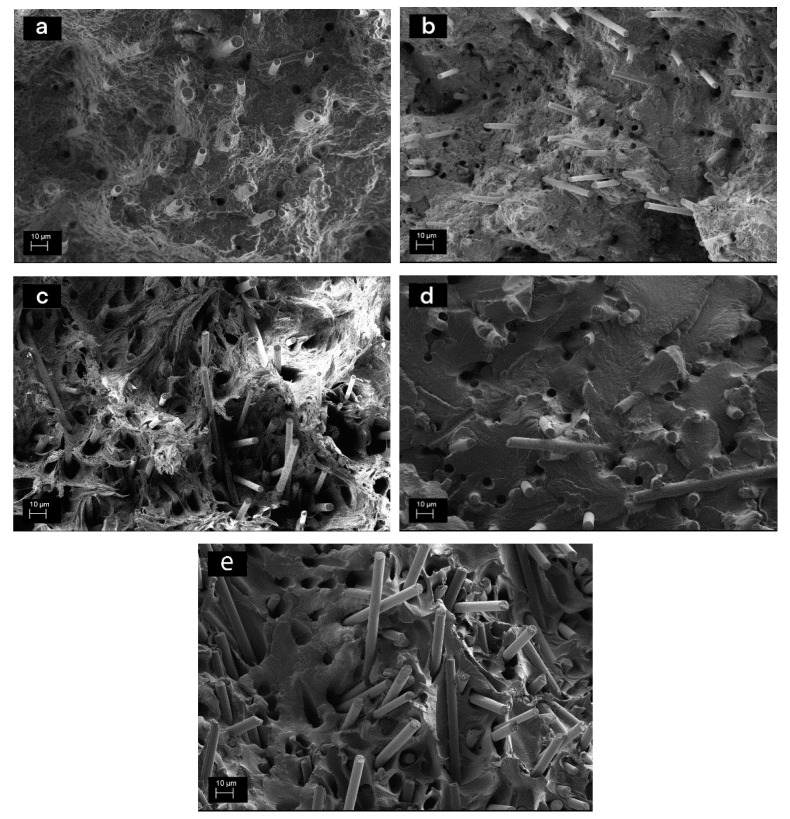
Fracture surfaces of (**a**) rCF-PP compound without any fibre surface treatments, (**b**) rCF-PP with 5-% sizing, (**c**) rCF-PP acid treatment with 5 w-% sizing, (**d**) rCF-PA6 without any treatment and (**e**) rCF-PA6 with 5-% sizing.

The fracture surfaces of the rCF compounds (Figure 8) reveal the same as the tensile tests: there are hardly any differences between samples with and without sizing. Apparently, the thermal recycling process leads to a favourable carbon fibre surface structure, and from an adhesion point of view, the sizing is not required. However, the acidic treatment seems to destroy the adhesion, which partly explains the poor tensile test results. The surface of the fibres is clean, and no matrix residues are visible. Acetone washing led to similar fracture surface with the corresponding samples without the pre-treatment.

### 3.5. The Efficiency of the Resizing

To evaluate the reinforcing efficiency of the resized fibres, the tensile properties of the thermoplastic compounds were compared with a theoretical ideal value. The rule of mixtures (RoM) is commonly used to approximate the tensile modulus and strength of fibre-reinforced polymer composites. RoM is based on the properties of the components as well as their relative volume fractions. The reinforcing efficiency of fibres is related to the load transfer at the fibre matrix interface. Many models exist for prediction of composite properties. One of the models to predict the tensile modulus (*E*_1_) of short, aligned glass and carbon fibre-reinforced composites is Halpin-Tsai equation [35]:

(1) $$E_{1}=E_{M}\left(\frac{1+\xi \eta V_{f}}{1-\eta \psi V_{f}}\right),$$

where the parameters *η* and *ψ* are given as:

(2) $$\eta =\frac{\left(\frac{E_{F}}{E_{M}}\right)-1}{\left(\frac{E_{F}}{E_{M}}\right)+\xi },$$

In the Equations (1)–(3), *E_F_* and *E_M_* are the tensile modulus of fibres and matrix, *V_f_* fibre volume fraction, *ξ* is a shape fitting parameter to fit the Halpin-Tsai equation to the experimental data, *ψ* depends upon the particle packing fraction and *φ*_max_ is the maximum packing fraction and has a value 0.82 for random packing of fibres. If fibres with a circular cross-section are oriented in the flow direction during compounding, *ξ* can be given for longitudinal modulus by the Equation (3):

(3) $$\xi =2\left(\frac{L}{D}\right),$$

where *L* refers to the length of a fibre in the flow direction and *D* is the diameter of the fibre.

The Halpin-Tsai model uses the following assumptions: the fibres are axisymmetric, identical in shape and size. The orientation of the fibres is assumed to be longitudinal and the distribution random. Additionally, the fibres should strongly bond to the matrix interface, and no interfacial slip during deformation is considered [36]. The recycled fibres in this case are symmetric, their length is approximately constant, and they are relatively well aligned due to the injection moulding processing. Thus, the Halpin-Tsai equation can be used here to evaluate the reinforcing efficiency of the fibres. The fibre volume fraction was 20 w-%, as stated in the Section 2.3. The approximate length of the fibres in the composite material after compounding was evaluated to be 0.1 mm, as compounding with a twin screw mixer shortens the fibres. The predicted elastic moduli for the composites are presented in Table 5. The best effect of resizing was achieved with 5 w-% PP-based sizing for rGF. The experimental results of the modulus were 19% lower than the predicted value, indicating very good reinforcing efficiency.

## 4. Conclusions

The aim of this study was the optimization of the batch-type sizing processes for recycled fibres. The effect of different sizing variables, such as pre-treatments of fibres, the concentration of the sizing solution, and rinsing the resized fibres after sizing, were tested for recycled glass and carbon-reinforced thermoplastics. Fibre surface pre-treatments were found to be neither needed nor successful to improve the final composite properties. Increasing the concentration of the sizing solution increased the amount of the sizing on the fibre surface whereas rinsing the fibres after the batch-type sizing decreased the amount of the sizing—both following intuitive behaviour. For glass fibres, an initial increase followed by a decrease in composite properties was found as a function of the amount of sizing. This trend was more distinct for the polypropylene-based sizing for polypropylene matrix than for the polyurethane-based sizing for polyamide matrix. The reason was analysed to be the plasticising effect of excess film former at the fibre–matrix interphase, highlighting the importance of the rinsing step in the process. For recycled carbon fibres, the sizing had little effect on the tensile properties of the thermoplastic composites, but the sizing was essential to gain better handling and post-processing properties. A comparison between experimental results and theoretical prediction using Halpin–Tsai model showed up to 81% reinforcing efficiency for glass fibres and up to 74% for carbon fibres.

## Figures and Tables

**Table 1:** Properties of the recycled glass (rGF) and carbon (rCF) fibres used in the study.

Fibre	Tensile Strength [MPa]	Modulus [GPa]	Length [mm]
rGF	447 ± 181	54 ± 12	50–300
rCF	1328 ± 494	102 ± 16	>300

**Table 2:** The rGF sizing solution formulations for 1 and 5 w-% solid content solutions and the related process steps together with the amount of sizing obtained from thermogravimetric analysis (TGA).

Sample	1st Mixing Step (1 h)			2nd Mixing Step (1 h)		Rinsing	Amount of Sizing [w-%]
	Coupling Agent [g]	Deionised Water [g]	Acetic Acid [g]	Film Former [g]	Deionised Water [g]		
rGF 1 w-% PP	0.10	50.00	0.10	2.25	47.55	Yes	0.14
rGF 1 w-% PP, no rinsing						No	4.61
rGF 5 w-% PP	0.50	50.00	0.50	11.25	37.75	Yes	0.70
rGF 5 w-% PP, no rinsing						No	4.32
rGF 1 w-% PU	0.10	50.00	0.10	1.50	48.30	Yes	0.55
rGF 1 w-% PU, no rinsing						No	1.23
rGF 5 w-% PU	0.50	50.00	0.50	7.50	41.50	Yes	0.53
rGF 5 w-% PU, no rinsing						No	7.01

The pH of all sizing solutions was adjusted to 7 with acetic acid.

**Table 3:** The rCF sizing solution formulations for 1 and 5 w-% solid content solutions and the related process steps together with the amount of sizing obtained from thermogravimetric analysis (TGA).

Sample	Film Former [g]	Deionised Water [g]	Solids Content in the Resizing Solution [w-%]	Rinsing	Amount of Sizing [w-%]
rCF 1 w-% PP	5	195	1	Yes	1.01
rCF 5 w-% PP	25	175	5	Yes	2.08
rCF 1 w-% PU	2	198	1	Yes	0.87
rCF 5 w-% PU	10	190	5	Yes	2.24

**Table 4:** Tensile test results for the rCF-PP and rCF-PA6 composites.

Sizing	Matrix	Tensile Strength [MPa]	Tensile Modulus [GPa]
rCF, no sizing	PP	66 ± 4	4.6 ± 0.5
rCF 1 w-% PP		65 ± 3	4.5 ± 0.3
rCF 5 w-% PP		65 ± 3	4.5 ± 0.5
rCF acetone washing		69 ± 2	5.0 ± 0.1
rCF acidic treatment 5 w-% PP		44 ± 3	4.7 ± 0.2
rCF, no sizing	PA6	114 ± 4	3.7 ± 0.5
rCF 1 w-% PU		132 ± 3	4.1 ± 0.7
rCF 5 w-% PU		123 ± 2	4.1 ± 0.6

**Table 5:** Tensile moduli of the composite materials predicted by the Halpin-Tsai equation. The reinforcing efficiency is calculated by comparing the theoretical and best experimental values.

Composite Type	Theoretical Tensile Modulus [GPa]	Achieved Reinforcing Efficiency [%]
rGF + PP	3.7	81.1
rGF + PA6	5.8	56.8
rCF + PP	6.8	73.5
rCF + PA6	10.4	39.4
